# Supplementary material for: Prefrontal-amygdalar oscillations related to social behavior in mice
Source: eLife. 2022 May 17;11:e78428. doi: 10.7554/eLife.78428 (PMC9113747; doi:10.7554/eLife.78428)
Supplement: Transparent reporting form [file elife-78428-transrepform1.docx]

***eLife’s* transparent reporting form**

We encourage authors to provide detailed information *within their submission* to facilitate the interpretation and replication of experiments. Authors can upload supporting documentation to indicate the use of appropriate reporting guidelines for health-related research (see [EQUATOR Network](http://www.equator-network.org/%20)), life science research (see the [BioSharing Information Resource](https://biosharing.org/)), or the [ARRIVE guidelines](http://www.plosbiology.org/article/info:doi/10.1371/journal.pbio.1000412) for reporting work involving animal research. Where applicable, authors should refer to any relevant reporting standards documents in this form.

If you have any questions, please consult our Journal Policies and/or contact us: [editorial@elifesciences.org](mailto:editorial@elifesciences.org).

**Sample-size estimation**

- You should state whether an appropriate sample size was computed when the study was being designed
- You should state the statistical method of sample size computation and any required assumptions
- If no explicit power analysis was used, you should describe how you decided what sample (replicate) size (number) to use

Please outline where this information can be found within the submission (e.g., sections or figure legends), or explain why this information doesn’t apply to your submission:

Concerning our analyses of LFP signals, we first elected to re-analyze our pre-existing dataset (Abe et al., 2019; n = 8 mice), which not only allowed us to reduce experimental animal use, but which also previously proved sufficient to resolve several behavior-relevant LFP signals (e.g. 20-40 Hz oscillation), suggesting sufficient statistical power to uncover detailed behavior-relevant LFP oscillations in this study with 14 mouse samples (i.e. 4-7 Hz and 30-60 Hz oscillations). Concerning our spike analyses, the following is included in the **Spike unit analysis** subsection of the **Materials and methods** section: “No *a priori* power analyses were performed to determine target sample sizes. Experiments were instead designed to encompass a comparable number of cells as several previous studies of spike-phase computation among prefrontal principal cells (e.g. Karalis et al., 2016; Abe et al., 2019; Okonogi & Sasaki, 2021).”

**Replicates**

- You should report how often each experiment was performed
- You should include a definition of biological versus technical replication
- The data obtained should be provided and sufficient information should be provided to indicate the number of independent biological and/or technical replicates
- If you encountered any outliers, you should describe how these were handled
- Criteria for exclusion/inclusion of data should be clearly stated
- High-throughput sequence data should be uploaded before submission, with a private link for reviewers provided (these are available from both GEO and ArrayExpress)

Please outline where this information can be found within the submission (e.g., sections or figure legends), or explain why this information doesn’t apply to your submission:

Biological replicates (i.e., cells recorded) are explicitly detailed within figures or, where results are stated without an accompanying figure, in the text of the **Results** section. Experimental/technical replicates (i.e., trials recorded per electrode and cell) are detailed in the **Electrophysiological recording** subsection of the **Materials and methods** section as follows: “All recordings from a behavioral task were performed once so that all the tasks were novel for the mice and no duplications of samples were thus included”. Concerning outliers, the **Data analysis** subsection of the **Materials and Methods** section details that: “No outliers were excluded from all analyses”. As specified in the **Electrophysiological recording** subsection of the **Materials and Methods** section: “video frames with massive optical noise or periods that were not precisely recorded due to temporal breaks of image data processing were excluded”. Otherwise, no data points (including data sample outliers) were excluded from analysis.

**Statistical reporting**

- Statistical analysis methods should be described and justified
- Raw data should be presented in figures whenever informative to do so (typically when N per group is less than 10)
- For each experiment, you should identify the statistical tests used, exact values of N, definitions of center, methods of multiple test correction, and dispersion and precision measures (e.g., mean, median, SD, SEM, confidence intervals; and, for the major substantive results, a measure of effect size (e.g., Pearson's r, Cohen's d)
- Report exact p-values wherever possible alongside the summary statistics and 95% confidence intervals. These should be reported for all key questions and not only when the p-value is less than 0.05.

Please outline where this information can be found within the submission (e.g., sections or figure legends), or explain why this information doesn’t apply to your submission:

Concerning the presentation of data and p-values, the **Statistical analysis** subsection of the **Materials and methods** section describes that: "For normally-distributed data, individual data points are displayed in addition to sample mean and standard errors of the mean or presented in the sourcedata.xlsx. For non-normally-distributed data, data are displayed as distributions, with data points presented in the sourcedata.xlsx”. Values in text are reported as mean ± standard errors of the mean, except where noted. Single and double asterisks in figures denote statistical significance at p<0.05 and p<0.01 levels, respectively. These are described in the Figure legends. Concerning statistical analysis methods, the **Statistical analysis** subsection of the **Materials and methods** section describes that: “For each statistical test, data normality was first determined by the F test, and non-parametric tests applied where appropriate.” Further, each statistical test applied is explicitly detailed in figure legends or, where results are stated without an accompanying figure, in the text of the **Results** section, with exact p-values given in all cases (including non-significant results) and effect sizes and degrees of freedom provided where appropriate (e.g., ttests etc.).

(For large datasets, or papers with a very large number of statistical tests, you may upload a single table file with tests, Ns, etc., with reference to sections in the manuscript.)

**Group allocation**

- Indicate how samples were allocated into experimental groups (in the case of clinical studies, please specify allocation to treatment method); if randomization was used, please also state if restricted randomization was applied
- Indicate if masking was used during group allocation, data collection and/or data analysis

Please outline where this information can be found within the submission (e.g., sections or figure legends), or explain why this information doesn’t apply to your submission:

All mouse groups were assigned based on their background (e.g., wild, Shank3KO, or defeated). No further distinguishing criteria were applied. Sample allocation of cell types is explicitly detailed throughout the **Results** section. No masking was possible in assigning cells/pairs to specific cell-type groups, as cell types were discriminated based on their firing rates and spike width (Fig. 6A).

**Additional data files (“source data”)**

- We encourage you to upload relevant additional data files, such as numerical data that are represented as a graph in a figure, or as a summary table
- Where provided, these should be in the most useful format, and they can be uploaded as “Source data” files linked to a main figure or table
- Include model definition files including the full list of parameters used
- Include code used for data analysis (e.g., R, MatLab)
- Avoid stating that data files are “available upon request”

Please indicate the figures or tables for which source data files have been provided:

All source data for main Figures Supplementary figures can be found in the additional file “sourcedata.xlsx”
